# Calorimetric monitoring data of the evolution of the lamellar-inverted hexagonal phase transition in phosphatidylethanolamine dispersions upon temperature cycling

**DOI:** 10.1016/j.dib.2018.03.056

**Published:** 2018-03-17

**Authors:** Rumiana Koynova, Boris Tenchov

**Affiliations:** a Northwestern University, Department Molecular Biosciences, Evanston, IL, USA; b Columbus, OH, Ohio State University College of Pharmacy Columbus, OH, USA; c Medical University-Sofia, Department Medical Physics and Biophysics, Sofia, Bulgaria

## Abstract

The data presented in this article are related to the research article entitled "Cubic phases in phosphatidylethanolamine dispersions: formation, stability and phase transitions**"** (Tenchov and Koynova, 2017) [1]. This article presents thermodynamic data obtained by differential scanning calorimetry following the evolution of the L_α_ - H_II_ endotherm upon temperature cycling during the lamellar to cubic phase conversion.

**Specifications Table**

**Table:** 

Subject area	Physical chemistry, Biology
More specific subject area	Lipid phase behavior
Type of data	Figures (DSC scans), graph
How data was acquired	Differential scanning calorimetry; high-sensitivity differential adiabatic scanning microcalorimeter DASM-4 (Biopribor, Pushchino, Russia)
Data format	Raw data collection and analysis
Experimental factors	Lipid dispersions were homogenized by vortex-mixing.
Experimental features	Temperature cycling of the samples was applied directly in the calorimetric cell.
Data source location	N/A
Data accessibility	Data are presented in this article.

**Value of the data**

• The present data provide for the first time thermodynamic information concerning the cubic phase formation in PE dispersions upon temperature cycling through their L_α_ - H_II_ transition.
• The data demonstrate that the L_α_ - H_II_ phase transition endotherm gradually decrease in enthalpy during the lamellar to cubic phase conversion upon *T*-cycling; the transition temperature exhibits specific, two-phase dependence on the cycle number.
• The cubic phases found to form in diluted DPoPE dispersions are of particular interest because of their facile formation and stability over prolonged periods of time at physiologically relevant conditions in a broad temperature range.

## 1 Data

**Fig. 1:**
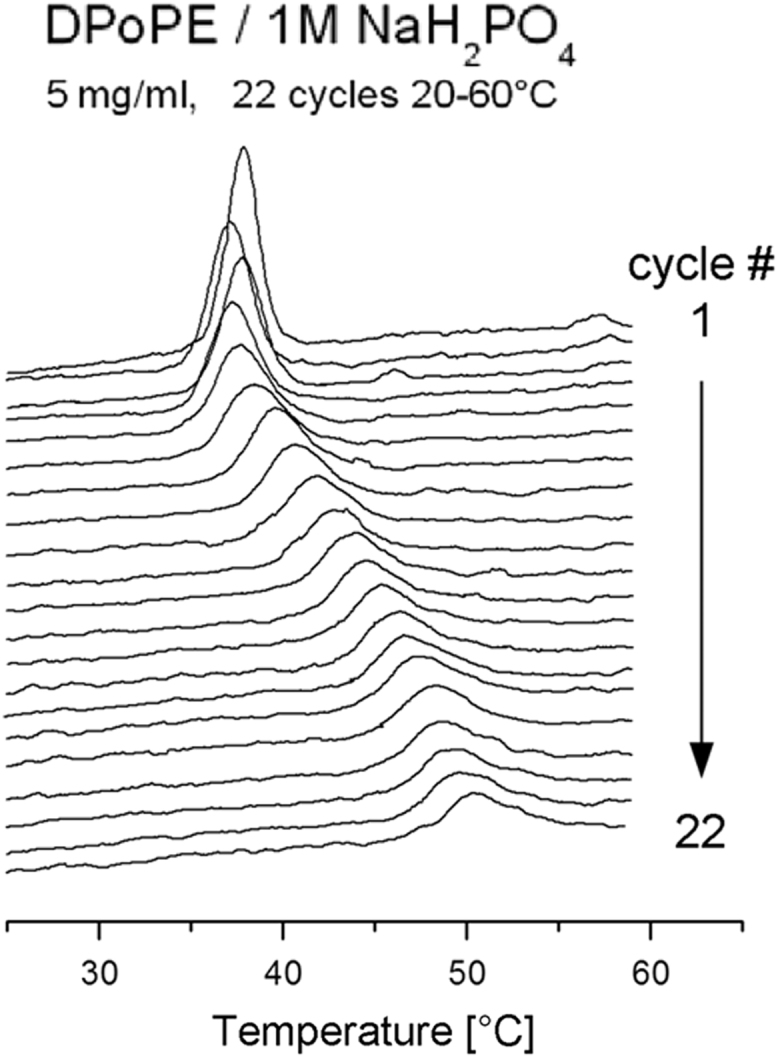
Consecutive calorimetric scans of the L_α_ → H_II_ transition in DPoPE / 1 M NaH_2_PO_4_ (5 mg/ml) dispersion recorded upon *T*-cycling 20–60 °C at 1 °C/min.

**Fig. 2:**
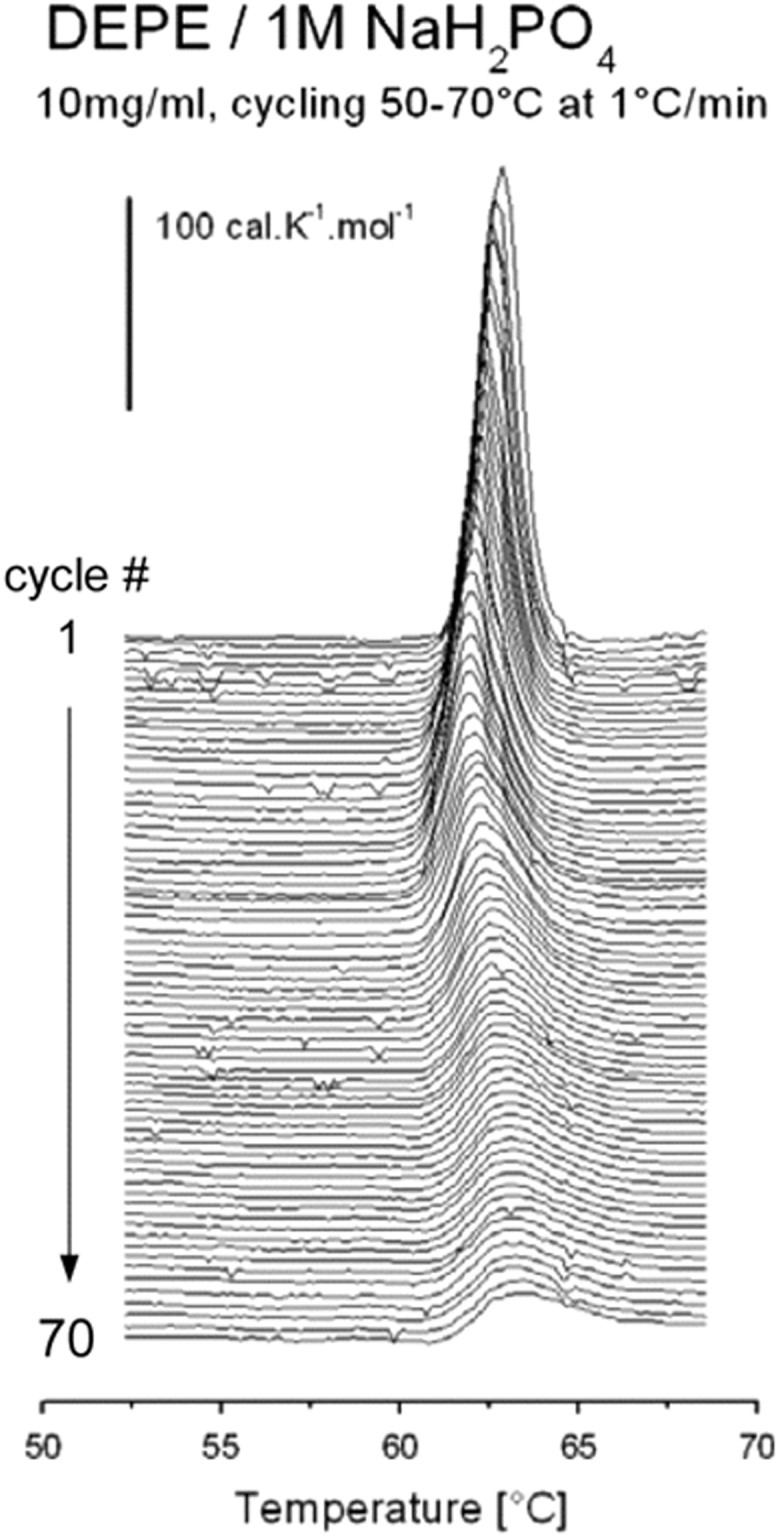
Consecutive calorimetric scans of the L_α_ → H_II_ transition in DEPE / 1 M NaH_2_PO_4_ (10 mg/ml) dispersion recorded during *T*-cycling 50–70 °C at 1 °C/min.

**Fig. 3:**
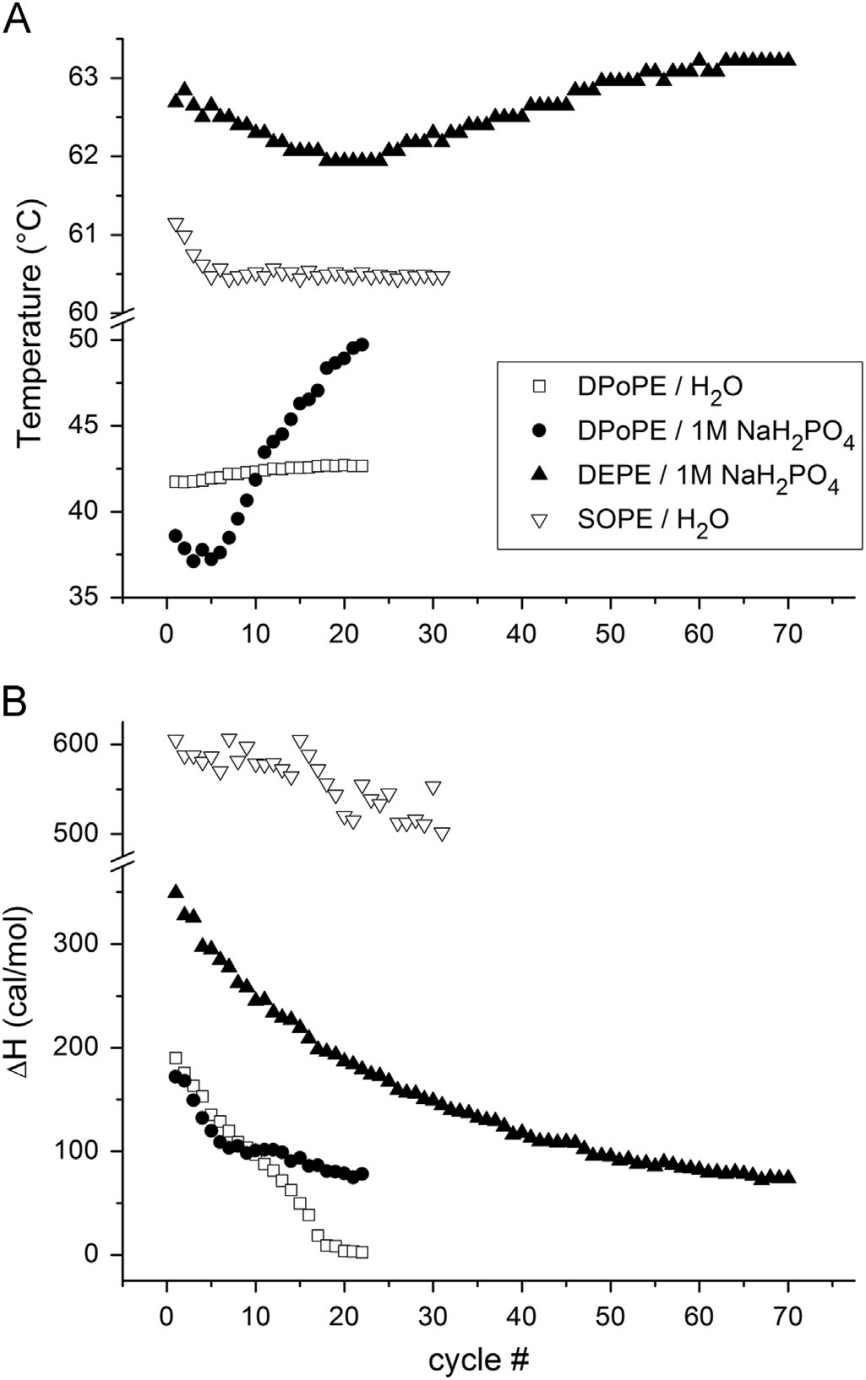
Evolution of the thermodynamic parameters of the L_α_-H_II_ transition in PE dispersions during *T*-cycling as a function of the cycle number: (A) transition temperature *T*; (B) transition enthalpy ΔH.

It has been reported that phosphatidylethanolamine (PE) dispersions are able to form highly stable Im3m and Pn3m cubic phases as a result of a temperature cycling through their L_α_ - H_II_ transition, as demonstrated by X-ray diffraction [1], [2]. The data presented here include calorimetric scans recorded while applying temperature cycling on PE dispersion samples directly in the calorimetric cell (Fig. 1, Fig. 2). Further, the thermodynamic parameters (temperature and enthalpy) of the recorded endotherms were calculated and plotted as a function of the cycle number (Fig. 3). The L_α_ - H_II_ phase transition endotherm gradually decrease in enthalpy during the lamellar to cubic phase conversion upon *T*-cycling. The transition temperature exhibits specific, two-phase dependence on the cycle number.

## 2 Experimental design, materials and methods

1,2-dipalmitoleoyl-sn-glycero-3-phosphoethanolamine (DPoPE), 1,2-dielaidoyl-sn-glycero-3-phosphoethanolamine (DEPE), and 1-stearoyl-2-oleoyl-sn-glycero-3-phosphoethanolamine (SOPE) were from Avanti Polar Lipids Inc. (Alabaster, AL). All lipids were found to migrate as single spot in thin-layer chromatography checks. Microcalorimetric scans of their diluted dispersions showed highly cooperative phase transitions at temperatures in agreement with the published values.

Samples were prepared by dispersing weighed amount of lipid into required amount of the appropriate solution. Samples of DPoPE were homogenized by vortex-mixing 8–10 times for 1–2 min at room temperature, at which they are in liquid-crystalline state. Samples of DEPE and SOPE, which are in gel phase at room temperature, were homogenized by cycling 8–10 times between 40 °C (above their chain-melting transition) and ice bath and vortex-mixed at these temperatures for 1–2 min. Lipid concentrations were 5–25 mg/ml.

Microcalorimetric measurements were performed using a high-sensitivity differential adiabatic scanning microcalorimeter DASM-4 (Biopribor, Pushchino, Russia) with sensitivity better than 4.10^–6^ cal K^−1^ and a noise level less than 5.10^–7^ W. Multiple heating–cooling cycles in temperature ranges similar to those used in the X-ray experiments were directly performed on samples in the calorimeter cell. Transition enthalpies and temperatures were determined in a standard way, as previously described [3].
