# Supplementary material for: Calorimetric monitoring data of the evolution of the lamellar-inverted hexagonal phase transition in phosphatidylethanolamine dispersions upon temperature cycling
Source: Data Brief. 2018 Mar 17;18:501–5. doi: 10.1016/j.dib.2018.03.056 (PMC5996298; doi:10.1016/j.dib.2018.03.056)
Supplement: Supplementary file 1 — Supplementary material [file mmc1.docx]

The authors declare no conflicts of interest.
